# Association between Presenteeism, Psychosocial Aspects of Work and Common Mental Disorders among Nursing Personnel

**DOI:** 10.3390/ijerph17186758

**Published:** 2020-09-16

**Authors:** Aline Silva-Costa, Pollyana C. S. Ferreira, Rosane H. Griep, Lucia Rotenberg

**Affiliations:** 1 Department of Collective Health, Federal University of Triangulo Mineiro, Uberaba 38025180, Brazil; pollyana.ferreira@uftm.edu.br; 2 Laboratory of Health and Environment Education, Oswaldo Cruz Institute, Fiocruz, Rio de Janeiro 21040900, Brazil; rohgriep@ioc.fiocruz.br (R.H.G.); rotenber@ioc.fiocruz.br (L.R.)

**Keywords:** presenteeism, common mental disorders, mental health, psychological demands, social support at work, psychosocial aspects of work, job stress, productivity loss

## Abstract

Presenteeism is the term used to describe going in to work even with a health problem. The phenomenon has been identified as one prior factor in sickness absence and, accordingly, the better it is understood, the better will be the prevention strategies. This study aimed to examine the mediating role of presenteeism (the ability to concentrate on work and to complete work despite a health problem) in the association between psychosocial factors at work and common mental disorders (CMD). This cross-sectional study included 1218 nursing personnel at a public hospital in Brazil. Structural equation modelling was performed. The sample comprised mostly women (85.4%), and the mean age was 44.1 (SD = 11.3; range: 24–70) years. Prevalence of presenteeism was 32.8%. Among presenteeist workers (*n* = 400), a relationship was observed between presenteeism and higher CMD scores. Furthermore, being able to concentrate on work even with a health problem mediated the relationship between social support and CMD and between psychological demands and CMD. Working when sick impairs both the work and the worker’s health. Interventions designed to improve working conditions and interpersonal relations can be effective strategies against presenteeism.

## 1. Introduction

Presenteeism is the term used for when people continue going to work, even with a physical or psychological health problem [1]. In such cases, the quality of the work performed may be impaired, entailing lost productivity and reflecting in losses to workers’ health, to the institution where they work [2,3] and to society [4]. Moreover, presenteeism has been considered an early indicator of future sickness absence and disability pensions [4]. Given that working while sick is linked to productivity loss, the stress related to lack of ability to fully perform work tasks can aggravate several chronic disease conditions. In addition, presenteeist workers do not stay home to take care of their health problem, which can further impair worker’s health. Thus, recurrent episodes of presenteeism may intensify the severity of the health problems, which may result in a longer sick leave [5,6,7]. In many situations, the presenteeist or absenteeist behavior might be related to the health problem affecting the worker [8]. This phenomenon is little-recognized, often hardly perceived and difficult for managers to identify [9].

Presenteeism has been associated with psychosocial factors at work [10,11]. High demands and hard effort, low levels of job control and reward at work, and low levels of social support received at work all play prominent roles in high rates of presenteeism. Shimabuku et al. [11] pointed out that greater job control strengthens autonomy in performing tasks; allied to stronger support from supervisors and co-workers, this enables workers to be more confident about missing work when faced with a health problem, thus reducing presenteeism.

In addition, longitudinal studies indicate that presenteeism is a risk factor for depression [12] and for mental wellbeing assessed on a scale including items related to depression and anxiety [13]. However, evidences are not conclusive due to the large variety of research methods and presenteeism measures (experience (yes/no), frequency and/or productivity losses) [14], which limit comparability [5]. Besides, the results might vary by country as in lower income countries, individuals with depression often do not report the disease to the employer in order to avoid dismissal, which result in lower levels of presenteeism reported by workers [15]. Therefore, differences within countries’ welfare systems and work policies are related to presenteeism and still need to be further explored in the literature [5].

Considering the above, it can be said that (i) psychosocial factors at work are associated with mental disorders [16,17,18], and (ii) presenteeism is associated both with psychosocial work factors [10,11] and with mental health [12,13]. This evidence from the literature raises the following question: can the association between psychosocial factors at work and mental health be mediated by presenteeism? Therefore, an empirical investigation, based on structural equation modelling, into the complex relationships among psychosocial factors at work, presenteeism and mental health constitutes a promising approach to exploring the possible mediating role of presenteeism. As presenteeism is a risk factor for sickness absence [19], the better the phenomenon is understood, the better will be the strategies developed to prevent sickness absence. 

When examining the relationships among presenteeism, psychosocial work environment and mental health, it is to be noted that nurses are among the personnel groups that often display presenteeism [3], as they may be exposed to the high levels of occupational stress, overwork, emotional demands of hospital work and insufficient rest time associated with occupational malaise [20,21,22,23]. In addition, a recent review on contextual and individual factors related to presenteeism pointed out that health professionals tend to show high overcommitment to work and a strong sense of responsibilities with patients, which prevent them from being absent from work, even if they are sick. Furthermore, for many healthcare workers, sick leaves can be faced as a sign of weakness [6]. A study on nurses’ perceptions of factors related to presenteeism stated that guilt was a feeling often reported by this group of workers. The increase of the team’s workload due to lack of professionals to assume the tasks, as well as the attempt not to disappoint coworkers, patients and family, are some of the reasons that lead nurses to attend work while sick [24].

The objective of this study was to examine the mediating role of lost productivity from presenteeism (the ability to concentrate on work and to complete work despite a health problem) in the association between psychosocial factors at work (psychological demands, skills discretion, decision authority and social support) and common mental disorders (CMD) among nursing workers at a public hospital.

## 2. Materials and Methods

This cross-sectional study of professional nursing personnel (nurses and nursing technicians and auxiliaries) from one of the largest federal public hospital in Rio de Janeiro, Brazil was conducted between December 2012 and May 2013. Each nursing worker was contacted personally by an interviewer who explained the aims of the study and invited them to participate. All those actively giving care were invited to take part (N = 1332). After refusals (*n* = 108; 8.1%), the study group totaled 1224 workers, from whom this study considered a sample of 1218 nursing workers, given that 6 participants did not answer the question about presenteeism. The total study population comprised mostly women (85.4%); 33.2% were registered nurses and 62.4% had completed higher education. Mean age was 44.1 (standard deviation [SD] = 11.3; range: 24–70) years. Respondents worked at the hospital for an average of 13.7 years (SD = 11.1; range = <1 year–43 years). Most participants (65.7%) worked ≤40 h/week, and 38.6% had a second job.

Data were collected at the hospital itself, during the nursing staff’s working hours, by applying a questionnaire on socio-demographic, work- and health-related information. The interviews were conducted by trained interviewers and all study quality assurance and control stages were duly fulfilled.

The study was approved by the research ethics committee (No. 635/11) and all participants signed a declaration of free and informed consent.

The variables included in the study were:

Presenteeism: evaluated by the question “In the past 30 days, have you come in to work despite having some health problem or sign or symptom of illness?”. Those who answered “Yes” were also evaluated on the Brazilian version of the Stanford Presenteeism Scale (SPS-6) [25], which assesses to what extent state of health may impair productivity, by measuring ability to concentrate and perform work despite a health problem [1]. The SPS-6 comprises six statements (1- hard to handle stress of my job; 2- able to finish hard tasks; 3- no pleasure in work; 4- hopeless about finishing certain tasks; 5- able to focus on achieving goals; 6- felt energetic enough to complete all the work) on a Likert-type scale, with five response options ranging from “strongly disagree” to “strongly agree”. The scores for statements 1, 3 and 4 were given by the sum of the responses, where “strongly disagree” equalled 5 points and “strongly agree” equalled 1 point. Statements 2, 5 and 6 were scored by “strongly disagree” corresponding to 1 point and “strongly agree”, to 5 points [3]. The statements make up two dimensions of the scale, with scores ranging from 3 to 15 points each. The “avoided distraction” dimension (statements 1, 3 and 4) assessed the ability to concentrate on work despite a health problem and the “work completed” dimension (statements 2, 5 and 6) addressed the ability to finish work despite a health problem. Thus, the higher the score, the greater the worker’s ability to concentrate on and complete all their work despite the health problem, i.e., higher scores indicated work less impaired by presenteeism or, put differently, less lost productivity [1,25]. Cronbach’s alphas were 0.759 and 0.713 for the dimensions avoided distraction and work completed, respectively.

Psychosocial work aspects: these were evaluated using the Brazilian version of the Demand-Control-Support Questionnaire [26]. The components of the scale are: (1) psychological demands, comprising five items (work fast, work intensively, work effort, time available and conflicting demands), (2) skills discretion, comprising three items (learning new things, skill and expertise and using initiative), (3) decision authority (how to do the work and what to do at work) and (4) social support at work, comprising six items (work environment, relations with co-workers, support from co-workers, co-workers’ understanding, relations with supervisor(s) and satisfaction with the team). The model of best fit was obtained by removing the “repetitive work” item from the “skills” component [27,28]. All the questions offered four response options on a Likert-type scale, ranging from “often” (score 1) to “never/nearly never” (score 4) and “strongly agree” to “strongly disagree” for the social support at work scale [26]. For each component, higher scores indicated higher levels of each dimension. Thus, the higher the score, the higher the levels of psychological demands, social support, skill discretion and decision authority. Cronbach’s alpha values were 0.815, 0.749, 0.562 and 0.676 for the social support, psychological demands, skills discretion and decision authority scales, respectively. Before removing the “repetitive work” item, the alpha was 0.426, reinforcing the need of removing this item in studies with nurses [27].

CMD: were measured by the self-reporting questionnaire (SRQ-20), comprising 20 dichotomous (Yes/No) questions that assessed anxiety, somatic and depressive symptoms displayed in the prior 30 days. Each affirmative response added one point to the final score, which could range from 0 (no likelihood of CMDs) to 20 (high likelihood of CMDs) [16,29]. Cronbach’s alpha was 0.815.

The covariables included were: sex, age (whole years), schooling (upper secondary; higher) and job category (nurse; nursing technician/auxiliary).

### Data Analysis

Descriptive analyses were performed to characterize the study population. The categorical variables were presented by absolute and relative frequencies, while the quantitative variables were described on the basis of means, standard deviations (SD), medians and interquartile range (IQR = P25%–P75%). Chi-square, Mann Whitney and Spearman correlation tests were performed.

**Figure 1:**
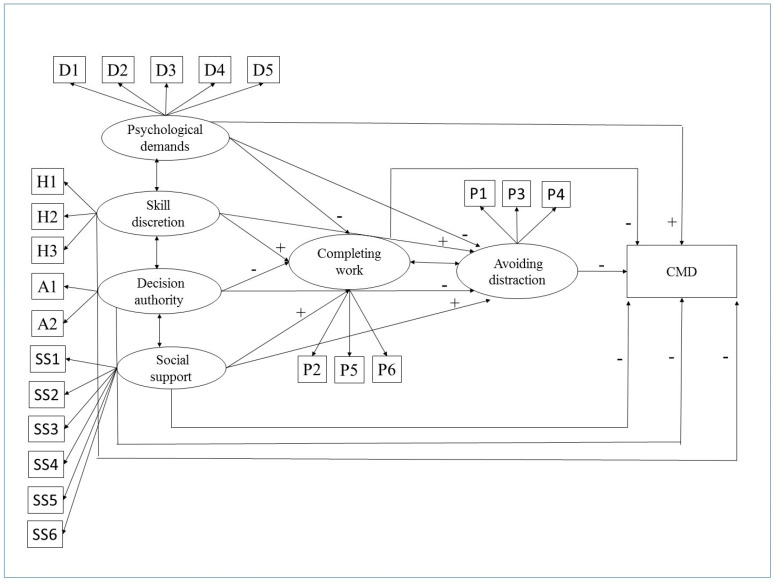
The role of presenteeism (work completed and distraction avoided despite health problem) in the relationship between psychosocial aspects of work and common mental disorders (CMD). Legend: D1: fast work, D2: intensive work, D3: work effort, D4: time available, D5: conflicting demands, H1: learning new things, H2: skill and expertise, H3: using initiative, A1: how to do work, A2: what to do at work, SS1: work environment, SS2: relationship with co-workers, SS3: support from co-workers, SS4: co-workers’ understanding, SS5: relationship with supervisor(s), SS6: satisfaction with team; P1: hard to deal with stress at work; P2: manage to complete difficult tasks; P3: no pleasure in work; P4: hopeless about completing some tasks; P5: manage to concentrate on goals; P6: energetic enough to complete all work. The signs (+ or -) refer to the expected associations between the variables.

Considering the group that reported going to work despite having a health problem (referred to here as “presenteeists”, *n* = 400), a structural equations model [30] was estimated relating psychosocial aspects of work, work completed, distraction avoided and CMD. According to the model hypothesized, the psychosocial aspects are associated with work impaired by presenteeism (distraction avoided and work completed), which are associated with CMD (Figure 1). Direct and indirect effects on CMD were estimated. The analyses were adjusted for age, sex and job category. The robust maximum likelihood method was used to estimate the parameters (standardised coefficients, expressed in SD). Model fit was assessed using the comparative fit index (CFI > 0.90), Root mean square error of approximation (RMSEA ≤ 0.05) with a 90% confidence interval (90%CI) and Tucker Lewis Index (TLI > 0.90) [31]. A level of significance of 5% was used in the statistical analysis, R software (version 2.15) (R Foundation for Statistical Computing, Vienna, Austria) and Mplus (version 7.4, Muthen & Muthen, Los Angeles, CA, USA) were used.

## 3. Results

### 3.1. Sample Description

In relation to the psychosocial aspects of work, the mean values were 13, 5 and 20 points for psychological demands, decision authority and social support dimensions, respectively (Table 1). The mean score for CMD was 4.94 (SD = 3.88) points, with a median of 4 (IQR = 2–8) points.

The prevalence of presenteeism was 32.8%. In the “distraction avoided” dimension (ability to concentrate despite a health problem), 50% of the group scored up to 8 points (IQR = 6–12), with the mean at 8.65 (SD = 3.78) points. In the “work completed” dimension, 50% of the group scored up to 13 points (IQR = 11–15), with a mean of 12.43 (SD = 2.97) points.

Table 2 shows that presenteeism was more frequent among the women, the workers facing higher levels of psychological demands, those scoring higher in CMD screening and those enjoying less social support (Table 2). Among the subgroup of presenteeist workers, CMD were found to correlate with the ability to concentrate on work (r = −0.300), the ability to complete work despite a health problem (r = −0.153), psychosocial demands (r = 0.202), social support (r = −0.324) and decision authority (r = −0.108), with statistical significance (*p* < 0.05). Correlation between CMD and skill discretion was not statistically significant (r = −0.029; *p* = 0.577).

### 3.2. The Mediating Role of Work Impairment in the Association Between Psychosocial Work Factors and CMD Among Presenteeist Nurses

For presenteeist workers, it can be seen that lower levels of social support and higher levels of psychological demands were associated with less ability to concentrate on work, i.e., the lower the social support and greater the psychological demands, the more presenteeism impaired the work (0.195; *p* = 0.012 and −0.239; *p* = 0.009, respectively). Furthermore, higher CMD scores were associated with less ability to complete work (−0.121; *p* = 0.025), lower level of concentration (−0.202; *p* < 0.001) and less social support (−0.224; *p* = 0.001). In other words, there was a direct relationship between work more impaired by presenteeism and higher CMD scores (Table 3).

Regarding the mediating role of work impaired by presenteeism, the ability to concentrate on work and not be distracted by health problems mediated the relationship between social support and CMD (−0.040; *p* = 0.039) and the relationship between psychological demand and CMD (0.048; *p* = 0.037) (Table 3). The factor loads for the latent variables were positive, with the following values: psychological demands (between 0.503 and 0.738), skill discretion (from 0.418 to 0.702), decision authority (0.699 and 0.710), social support (between 0.387 and 0.783), completing work (from 0.431 to 0.823) and avoiding distraction (between 0.628 and 0.761). The model returned good indices of fit: CFI = 0.93, RMSEA = 0.043 (90%CI: 0.036; 0.050), TLI = 0.91 and SRMR = 0.055. Analysis by job categories showed similar results.

## 4. Discussion

Considering the sample of presenteeist nursing personnel, the results of this study suggest that work impaired because of presenteeism mediated the association of heavy psychological demands and low social support with CMD. High psychological demands associated with decreased ability to concentrate on work because of a health problem (work impaired by presenteeism) were related to higher CMD scores. Similarly, low social support was associated with decreased ability to concentrate on work, which led to higher CMD scores. Note that although decision authority was associated with work impaired by presenteeism (decreased ability to complete the work), no statistically significant association was observed between decision authority (neither skill discretion nor psychosocial demands) and CMD. Therefore, a total statistically significant effect on CMD was found just from social support.

Baeriswyl et al. [32] observed that the characteristics of the work environment, social support and workload were important predictors of presenteeism and emotional exhaustion, and pointed to presenteeism as a mediating variable in the relationship between work characteristics and emotional exhaustion. Another study [33] showed that a number of factors related to high job stress are also related to greater presenteeism, in terms of both how often presenteeism occurs and lost productivity related to health problems (work impaired due to presenteeism). In the opposite direction from the model proposed in the study reported here, they assumed that both physical and mental health mediated the relationship between the areas of work life and presenteeism [33]. Similarly, Li et al. [34] also showed a relationship between job stress and presenteeism mediated by mental health. Nonetheless, few studies have assessed the role of work impaired by presenteeism in the relationship between psychosocial factors at work and mental health, which underlines the contribution offered by this study. As previous noted [33], continuing to go in to work despite illness prevents or hampers recovery, which in the long run can lead to higher rates of absence and more severe consequences from both lost productivity and worsening health problems.

Rainbow [24], using a qualitative approach, described factors leading to nurse presenteeism. The author noted that nurses often prioritize hospital needs over their own health. Given the sense of responsibility towards co-workers and patients, nurses states that when there is no one else to assume the work, it is preferable to go to work with reduced performance capacity rather than not having anyone in the unit. In addition, depending on the disease, sick absence is not well faced by the employer. Factors of personal life, such as the need to save days due to the possibility of family emergencies or for travel, are also considered. Finally, depending on organizational policy, absences can also result in loss of wages, which, therefore, would be avoided whenever possible by workers [24].

As many authors have argued, heavy psychological demands and poor social support are stressful conditions strongly associated with a number of adverse health outcomes [35,36,37,38]. Regarding presenteeism, our study suggests that perceptions of poor social support and heavy psychological demands are associated with lost productivity from presenteeism. A study with Korean workers found associations between several psychosocial factors and the occurrence of presenteeism [39]. Lack of support from co-workers, stress and the intensity of the work were some of the factors associated with presenteeism. However, they observed no significant association between presenteeism and support from supervisor(s) or autonomy at work [39]. In our study, we evaluated support from co-workers and from supervisors as a single group, which did not allow us to analyze possible differences between support from co-workers and from supervisors. Our results established the relevance of social support as a possible factor to avoid the presenteeism and the loss of productivity among presenteeist workers. Besides, in our study, high decision authority was related to decreased ability to complete the work. This result suggests that the possibility of choosing how to do the work and what to do at work allows for better management of tasks by workers when sick.

Stress was also associated with presenteeism among workers at a United States hospital [40]. It is thus plausible to suppose that the relationship between high psychological demands and presenteeism is framed by a sense of responsibility and strong commitment on the worker’s part. As observed here, approximately one third of the nursing personnel interviewed mentioned working despite a health problem. The fact that this group of workers deals with health, and directly with patient care, may explain this high level of presenteeism, at least in part. However, in order to assure quality care, it is essential that health personnel are willing and able in their work environment. That discussion contributed to an understanding of the relationship between presenteeism and low levels social support and, because the recognition that co-workers are supportive can relieve pressure and/or concern over disappointing them by needing to miss work because of a health problem [32,41]. In the study reported here, poor social support was found to be associated with going in to work despite a health problem, with consequent loss of productivity. Therefore, as proposed by Whysall et al. [8], presenteeism is more likely to occur in work environments where workers experience little social support (from supervisor(s) or from co-workers). A work environment with social support is related to a better health, which would lead to few cases of presenteeism. In addition, in general, supportive co-workers and supervisors might encourage employees to stay home while ill [42]. On the other hand, helpful colleagues may also explain the association between high social support and less lost productivity observed among presenteeist workers. In this regard, future interventions directed to reducing presenteeism or lost productivity related to presenteeism should consider the importance of social support in the workplace. As proposed by Li et al. [3], promoting a more supportive work environment, considering the quality of performance rather than the frequency of work attendance, can be a strategy to deal with presenteeism.

Regarding the relationship between psychosocial aspects of work and CMD, a meta-analysis of longitudinal studies [43] showed CMD to be associated with low control, high psychological demand and low social support at work. A study of health workers [17] also found statistically significant associations. However, they assessed the psychosocial aspects of work by quadrants of the demand-control model, unlike the study reported here, which considered the isolated effect of each component of the demand-control questionnaire. They found higher prevalence of CMD in workers under heavy demands or with poor social support at work. The association was strongest among those exposed to both heavy demands and poor social support [17]. A meta-review that examined the relationship between psychosocial factors and development of CMD, focusing on depression and anxiety, identified high psychological demand, low social support, bullying, work hours and other factors as risks for CMD [18].

Presenteeism runs counter to the logic that sick workers need rest in order to recover. Thus, in the long run, presenteeism may lead to a worsening of existing conditions or to the development of other physical and psychological problems. Specifically regarding mental health, the accumulated fatigue associated with symptoms of stress can trigger or potentiate symptoms of depression, anxiety and others [11]. In agreement with this, the study reported here found that the greater the presenteeism (including difficulty in concentrating and completing tasks because of a health problem), the higher the CMD scores.

Although our data was collected before the COVID-19, considering the current scenario related to the pandemic, the idea of working while sick deserves attention. A systematic review of presenteeism in relation to infectious disease points out that working with symptoms of acute infections like the flu or a cold can result in serious problems, given the possibility of epidemics in the workplace [44]. Workers usually differentiate between the types of health conditions they would take sick leave. One of the criteria adopted by nurses in order to decide to attend work when sick is non-contagious illness [24]. However, a study with healthcare workers found that 41% of the group reported having worked while experiencing influenza-like illness [45]. Generalizing to the current moment and recognizing the huge public health problem due to the rapid spread of the coronavirus, presenteeism must not occur even if the symptoms of the disease are mild. On the other hand, returning to the scenario of non-transmissible diseases, health professionals have frequently experienced work while unwell during the COVID-19 pandemic [46], in which presenteeism is linked to other problems such as fatigue, stress, exhaustion, lack of sleep, anxiety and depression [47,48]. Furthermore, in this scenario, social support remains an important coping strategy to reduce the work impaired by presenteeism [48].

Despite that the data from the current study was not collected recently, which needs to be addressed as an important limitation, the debate related to presenteeism is timely and deserves to be highlighted. In this perspective, it is relevant to mention that our results are in the same direction of those using more recent data. A study on factors associated with work performance during presenteeism in a cohort of nurses found a positive relationship between manager support and lower productivity loss [49]. Furthermore, nurses’ presenteeism was related to a decline in mental health [3]. In addition, low level of social support is associated with presenteeism [41]. These recent findings in agreement with our study stress the representativeness of our sample for the current model. Moreover, the profile of the studied sample did not change over time. In line with other nurse studies, we also observed a predominantly female population, referring to long work hours, high overload and complaints related to psychosocial stress [24,49,50,51]. Even so, our findings should be interpreted with caution, considering different work settings, professions and in particularly the current situations in hospitals.

We evaluated nurses’ professionals as a single group, as in several aspects (prevalence of CMD and presenteeism), we did not observe differences between nurses and nursing technicians. However, especially for psychological demands the association can be higher for nurses, considering that responsibilities at work are not the same. Besides, our sample is mostly composed of women, which should be taken into account. Finally, given the cross-sectional nature of the data, causal relationships could not be established. Although we have adopted a directionality from psychosocial factors to CMD mediated by lost productivity from presenteeism, it is possible that CMD might affect how nurses experience social support at work and difficulties in concentrating on work tasks.

## 5. Conclusions

This study showed that presenteeism is related to heavy psychological demands, poor social support and to CMD. Among presenteeist nursing personnel, lost productivity from presenteeism mediated the association of heavy psychological demands and social support with CMD. Going to work despite a health problem impairs both the work itself and the worker’s health. From the standpoint of job-related factors, interventions seeking to improve working conditions and interpersonal relations can be effective strategies against presenteeism. In addition, assessment of presenteeism, allied to strategies to prevent its occurring, can reduce productivity losses at work and CMD.

Interventions to reduce psychosocial stress at work may contribute to reducing health problems and, consequently, lost productivity from presenteeism. As mentioned above, managers should promote a supportive work environment, prioritizing the quality of care and avoiding punishments related to any sickness absenteeism. Besides, staff management policies should avoid working with a reduced number of nursing professionals, taking into account the possible occurrence of sickness absenteeism. This strategy could attenuate the feeling of guilt for overloading colleagues in the face of absence. Moreover, the increased number of nursing professionals can lead to a decrease in work demands, which would also diminish work-related health problems.

Future researches may consider a longitudinal design to examine the health problems that more often predict presenteeism in order to improve specific interventions in the workplace, and to better evaluate the effects of working while sick among nursing professional. Furthermore, considering that workloads would be different among hospital wards, future studies should explore this approach.

## Figures and Tables

**Table 1:** Assessment of dimensions of psychosocial aspects of the work of nursing personnel – Brazil, 2012–2013.

Dimensions of Psychosocial Aspects of Work	Min–Max	Mean	SD	Median	IQR
Psychological demands	5–20	13	2.90	13	11–15
Skills discretion	3–12	10.33	1.65	11	9–2
Decision authority	2–8	5	1.56	5	4–6
Social support	8–24	20	3.09	20	18–20

Min–max = minimum and maximum score values. IQR = interquartile range (P25%–P75%); SD = standard deviations.

**Table 2:** Sociodemographics, psychosocial work factors and common mental disorders among nursing personnel—Brazil, 2012–2013.

		Presenteeist Workers		
	Total Sample	No (*n* = 818)	Yes (*n* = 400)	*p*
		*n* (%)		
**Sex**				
Female	1042 (85.6)	684 (83.6)	358 (89.5)	0.006
Male	176 (14.4)	134 (16.4)	42 (10.5)	
**Schooling**				
Secondary	456 (37.4)	313 (38.3)	143 (35.8)	0.395
Higher	762 (62.6)	505 (61.7)	257 (64.3)	
**Job category**				
Nursing auxiliary/technician	813 (66.7)	552 (67.5)	261 (65.2)	0.438
Nurse	405 (33.3)	266 (32.5)	139 (34.8)	
		Mean (SD)		
**Psychological demands**	13.16 (2.89)	12.94 (2.90)	13.62 (2.82)	<0.001
**Skill discretion**	10.33 (1.65)	10.32 (1.68)	10.37 (1.59)	0.767
**Decision authority**	5.14 (1.56)	5.14 (1.59)	5.13 (1.52)	0.746
**Social support**	19.84 (3.09)	20.14 (2.93)	19.22 (3.31)	<0.001
**Common mental disorders**	4.95 (3.87)	4.19 (3.66)	6.49 (3.85)	<0.001
**Age**	44.06 (11.26)	44.06(11.53)	44.07 (10.71)	0.984

**Table 3:** Associations between psychosocial aspects of work, work impaired by presenteeism and common mental disorders, among presenteeists nursing personnel (*n* = 400)—Brazil, 2012–2013.

	Standardised Estimates (95%CI)	
***Direct Effects***		
Psychological demands → WC	0.035	(−0.141; 0.210)
Skills discretion → WC	0.042	(−0.133; 0.217)
Decision authority → WC	−0.183	(−0.326; −0.040) *
Social support → WC	0.113	(−0.046; 0.272)
Psychological demands → AD	−0.239	(−0.417; −0.060) *
Skills discretion → AD	0.123	(−0.052; 0.299)
Decision authority → AD	−0.012	(−0.156; 0.132)
Social support → AD	0.195	(0.043; 0.348) *
Psychological demands → CMD	0.093	(−0.057; 0.243)
Skills discretion → CMD	0.015	(−0.128; 0.158)
Decision authority → CMD	−0.054	(−0.179; 0.071)
Social support → CMD	−0.224	(−0.351; −0.097) *
WC → CMD	−0.121	(−0.227; −0.015) *
AD → CMD	−0.202	(−0.315; −0.090) *
***Indirect Effects***		
Psychological demands → AD → CMD	0.048	(0.003; 0.094) *
Skills discretion → AD → CMD	−0.025	(−0.063; 0.013)
Decision authority → AD → CMD	0.002	(−0.027; 0.032)
Social support → AD → CMD	−0.040	(−0.077; −0.002) *
Psychological demands → WC → CMD	−0.004	(−0.026; 0.017)
Skills discretion → WC → CMD	−0.005	(−0.027; 0.016)
Decision authority → WC → CMD	0.022	(−0.004; 0.048)
Social support → WC → CMD	−0.014	(−0.036: 0.009)
***Total Effect (Direct + Indirect)***		
Psychological demands → CMD	0.137	(−0.013; 0.288)
Skills discretion → CMD	−0.015	(−0.161: 0.131)
Decision authority → CMD	−0.030	(−0.155; 0.096)
Social support → CMD	−0.277	(−0.405; −0.150) *

* *p* < 0.05.
